# Host breed and geography shape the antiviral defense landscape of the bovine rumen microbiome

**DOI:** 10.1093/ismeco/ycag162

**Published:** 2026-06-11

**Authors:** Camila A Faleiros, Osiel S Gonçalves, Alanne T Nunes, Crislaine S Pires, Mirele D Poleti, Heidge Fukumasu

**Affiliations:** Department of Veterinary Medicine, School of Animal Science and Food Engineering (FZEA), University of São Paulo, Avenida Duque de Caxias Norte, 225—Jardim Elite, Pirassununga, SP 13635-900, Brazil; Department of Veterinary Medicine, School of Animal Science and Food Engineering (FZEA), University of São Paulo, Avenida Duque de Caxias Norte, 225—Jardim Elite, Pirassununga, SP 13635-900, Brazil; Department of Biological Science, Microbial Eco-Evolutionary Genomics Group, Midwestern Parana State University (UNICENTRO). Campus CEDETEG, Alameda Élio Antônio Dalla Vecchia, 838—Vila Carli, Guarapuava, PR 85040-167, Brazil; Department of Veterinary Medicine, School of Animal Science and Food Engineering (FZEA), University of São Paulo, Avenida Duque de Caxias Norte, 225—Jardim Elite, Pirassununga, SP 13635-900, Brazil; Department of Veterinary Medicine, School of Animal Science and Food Engineering (FZEA), University of São Paulo, Avenida Duque de Caxias Norte, 225—Jardim Elite, Pirassununga, SP 13635-900, Brazil; Department of Veterinary Medicine, School of Animal Science and Food Engineering (FZEA), University of São Paulo, Avenida Duque de Caxias Norte, 225—Jardim Elite, Pirassununga, SP 13635-900, Brazil; Department of Veterinary Medicine, School of Animal Science and Food Engineering (FZEA), University of São Paulo, Avenida Duque de Caxias Norte, 225—Jardim Elite, Pirassununga, SP 13635-900, Brazil

**Keywords:** antiviral defense systems, CRISPR-Cas, Nellore cattle, phage–host interactions, rumen microbiome

## Abstract

The rumen microbiome represents a complex, phage-rich ecosystem where microbial survival depends on both metabolic cooperation and antiviral defense. However, global and breed-associated variations in rumen prokaryotic immune systems remain poorly understood. Here, we performed the most comprehensive profile to date of antiviral defense systems (DS) in the rumen, analyzing 6530 microbial genomes and metagenome-assembled genomes (MAGs) from diverse cattle breeds and geographic regions. In this global dataset, we identified >90 000 DS, the most abundant of which were restriction-modification, PDC-S01, deoxyribonucleic acid modification systems (DMS_other), AbiE and SoFic, with variations influenced by both host the lineage and geographic region. A more in-depth analysis was performed using two complementary antiviral annotation frameworks for Nellore cattle (*Bos indicus*) from Brazil. Data exhibited a remarkably enriched antiviral defense repertoire, with over 15 632 DS encoded across 547 high-quality MAGs. These systems were densely clustered in dominant rumen lineages, such as *Prevotella*, and positively correlated with prophage abundance, consistent with virus–host coevolution. Notably, we also detected viral contigs encoding both antiviral defense and anti-defense genes, underscoring the arms race between the phages and their microbial hosts. Metatranscriptomic data from North America and Oceania revealed high expression levels of toxin-antitoxin modules, clustered regularly interspaced short palindromic repeats components, and restriction enzymes, suggesting a basal level of antiviral activity. These findings reveal the rumen as an antiviral innovation hotspot, highlighting microbiome resilience with implications for ecology, adaptation, and phage-based interventions.

## Introduction

The bovine rumen is a highly specialized and dynamic microbial ecosystem shaped by evolutionary pressures to maximize the degradation of complex plant polysaccharides and energy extraction from fibrous diets [1–3]. This anaerobic chamber harbors a complex assemblage of bacteria, archaea, protozoa, fungi, and viruses, collectively forming the rumen microbiome, which contributes up to 70% of the host’s energy via microbial fermentation [4–6]. Beyond its nutritional function, the ruminal microbiota is tightly linked to economically relevant traits in livestock production, including feed efficiency, weight gain, and methane emissions [7–12]. These traits are associated not only with taxonomic profiles but also with the functional repertoire encoded in microbial genomes, such as carbohydrate-active enzymes, stress response modules, and immune-related pathways [13–15].

Despite substantial research on rumen microbial metabolism, the virome represented by the bacteriophage and archaeal virus communities remains poorly characterized. Metagenomic surveys have uncovered vast viral novelty in the rumen, with large fractions of viral sequences lacking known homologs [16, 17]. These viruses can influence microbial dynamics by transferring auxiliary metabolic genes, impacting functions such as carbohydrate metabolism, amino acid biosynthesis, vitamin pathways, and methanogenesis [18, 19]. Furthermore, prophages, phage genomes integrated into bacterial chromosomes, are pervasive in rumen genomes, suggesting long-term virus–host coevolution and potential mutualism [20].

To persist in the face of intense phage predation, rumen microbes rely on a diverse array of antiviral defense systems (DS), collectively termed the prokaryotic immune system. These systems include well-established mechanisms such as restriction-modification (RM) and clustered regularly interspaced short palindromic repeats (CRISPR)-Cas, as well as recently discovered systems including Septu, Shango, AbiE, and CBASS, which employ molecular strategies ranging from nucleic acid sensing to abortive infection and metabolic sabotage [21–23]. Importantly, the DS are often distributed across community members, forming a “distributed immunity” that reinforces microbial resilience [24]. In response to DS, viruses have also developed immune strategies such as anti-CRISPR proteins and many others, which make up anti-defense mechanisms [25, 26].

**Figure 1:**
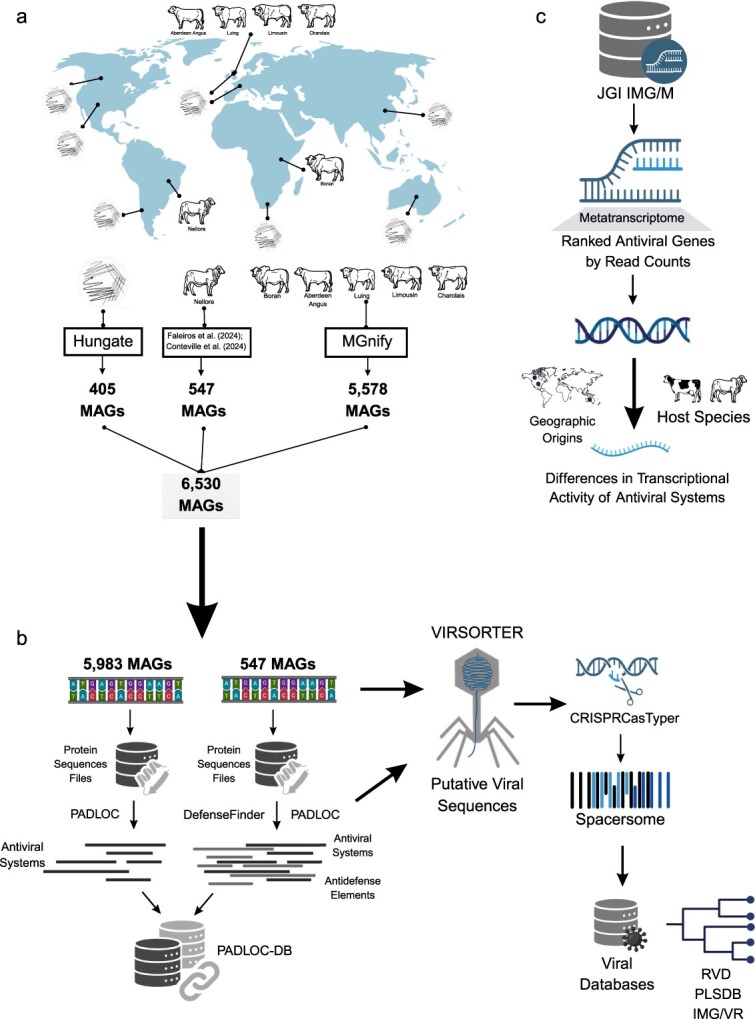
Experimental design. (a) Geographical location of MAGs distributed across three databases: Hungate, containing ruminal bacterial isolates; MGnify, with MAGs from the Boran, Aberdeen Angus, Luing, Limousin, and Charolais breeds; data from the Nellore breed originated from studies conducted in Brazil, totaling 6530 MAGs. PADLOC was employed to annotate the antiviral DS in rumen-derived MAGs. (b) A more in-depth analysis was applied to MAGs from the Nellore breed, which were subjected to DefenseFinder for DS annotation and analysis of anti-defense elements. The 547 MAGs were analyzed using VirSorter2 to identify putative viral sequences and to determine CRISPR-based DS using Spacersome and alignment with viral databases. (c) The JGI IMG/IM database was used for transcriptomic analysis, in which antiviral genes were ranked, demonstrating their expression in the rumen.

Although environmental factors and host phylogeny are known to shape virus–host interactions and the distribution of DS in the rumen [27], no study to date has systematically compared antiviral repertoires across cattle breeds and geographic contexts. We analyzed 6530 global rumen genomes to determine how host lineage and environment shape microbial antiviral repertoires (Fig. 1). Despite a smaller genome count, Brazilian Nellore cattle (*Bos indicus*) exhibited uniquely high-density antiviral systems, suggesting a breed-enriched immune landscape. Consequently, the Nellore microbiome was selected as a case study for compact yet functionally rich antiviral architecture within a global framework.

## Materials and methods

### Comparative dataset construction of bovine rumen microbial genomes

To establish a comprehensive genomic framework for exploring antiviral DS in the bovine rumen, a curated dataset of 5983 high-quality metagenome-assembled genomes (MAGs) derived from rumen samples across multiple cattle breeds and geographic regions, including: (i) the Cow Rumen Genome Catalogue v1.0, accessible via MGnify (5174 MAGs) generated from rumen metagenomic datasets [28]; (ii) 404 MAGs from African cattle [29]; and (iii) 405 reference genomes of isolates from the Hungate1000 Collection [5]. Only rumen-derived genomes were retained. Metadata on cattle breed, geographic origin, and study descriptors were extracted from the original publications for downstream comparative analyses (Supplementary Table S1).

As a focal subset, 547 high-quality MAGs from Nellore cattle (*B. indicus*), in Brazil. The genomes were retrieved from two publicly available studies [30, 31]. The dataset comprised high-quality genomes previously filtered based on CheckM [32] metrics (≥80% completeness and ≤10% contamination, with a final quality score ≥50) (Supplementary Table S1). Taxonomic classification of MAGs, was performed using the GTDB-Tk toolkit (v2.4.0) against the Genome Taxonomy Database (GTDB) release R220 [33]. The final dataset comprised 6530 rumen-derived MAGs. Notably, the taxa reported reflect the lowest informative taxonomic level reached per MAG. Further details are provided in the Supplementary Text 1.

### Annotation and identification of antiviral defense systems in Rumen metagenome-assembled genomes

To characterize antiviral defense in the bovine rumen microbiome, we analyzed 6530 high-quality MAGs. Annotation was performed using Prokka v1.14.6 [34], generating protein sequence files (*.faa) for downstream defense system prediction.

DS were identified using PADLOC v2.0.0 (Prokaryotic Antiviral Defence LOCator) [35], which employs Hidden Markov Models (HMMs) to identify canonical, novel antiviral systems, and catalog of experimentally validated and candidate defense *loci*. This enabled the detection of antiviral mechanisms, including well-characterized systems and recently described or underexplored systems, such as phage defense candidate (PCD) *loci*. To validate the predictions, all the detected systems were cross-checked against the PADLOC-DB reference database.

### Annotation and identification of antiviral defense systems in Nellore metagenome-assembled genomes

We analyzed 547 high-quality MAGs to characterize the antiviral defense repertoire encoded in the rumen microbiome of Nellore cattle. Protein sequence files (*.faa) were used as inputs for defense system detection using two complementary tools PADLOC v2.0.0 [35] and DefenseFinder v2.0.0 [36]. Predictions were validated against the PADLOC-DB reference database and DefenseFinder Wiki. Phylogenomic classification was performed using GTDB-Tk v2.4.0 (GTDB R220) [33] based on 120 bacterial and 53 archaeal conserved marker genes. The reconstruction was performed using IQ-TREE v1.6.11 [37] and visualized using the Interactive Tree of Life platform [38]. Further details are provided in the Supplementary Text 1.

### Detection of putative viral elements and identification of antiviral systems in rumen metagenome-assembled genomes from Nellore

The objective of this analysis was to detect and quantify putative viral sequences embedded within bacterial MAGs to investigate the relationship between viral load and host-encoded antiviral DS. We analyzed 547 high-quality MAGs derived from Nellore rumen, which were screened using VirSorter2 (v2.2.3) [39]. Only sequences containing ≤2 viral hallmark genes were retained. Quality and completeness were evaluated with CheckV (v1.0.1) [40]. Curation followed a stringent protocol based on the VirSorter2 SOP, a full description is provided in the Supplementary Text 1.

### Detection and characterization of clustered regularly interspaced short palindromic repeats–Cas systems and spacer matching against viral and plasmid databases

CRISPR-Cas systems were identified in rumen MAGs using CRISPRCas-Typer [41], which detects arrays, Cas genes, and predicts subtypes. Only the genomes with confirmed Cas systems were included. To determine the potential viral and plasmid origins of the spacers, we conducted similarity searches against four comprehensive reference datasets using blastn in a local environment: (i) the IMG/VR viral spacer BLAST database, a curated nucleotide viral [42]; (ii) PLSDB plasmid database (version 2024_05_31_v2) [43], comprising a broad collection of plasmid sequences; (iii) local viral genome database constructed from putative vMAGs obtained from rumen MAGs; and (iv) Rumen Virome Database (RVD) [18], a specialized database representing the rumen viral community. Significant spacer matches were defined as those with e-values of ≤1e-5. Viral taxonomic information was retrieved from IMG/VR and RVD metadata. Details, including statistics, are in the Supplementary Text 1.

### Metatranscriptomic analysis

To assess antiviral gene expression, metatranscriptomic datasets were retrieved from the JGI IMG/M. The study included rumen fluid metatranscriptomes from three locations: (i) UC Davis, California, USA; (ii) Lethbridge, Alberta, Canada; and (iii) Woodstock, Queensland, Australia. RNASeq expression data were obtained, and antiviral defensome genes were selected. Statistical analyses were performed in R. Counts were normalized using the DESeq2 package with the Relative Log Expression (RLE), followed by variance stabilizing transformation (VST). Further details are provided in the Supplementary Material (Table S1).

### Statistical analysis and visualization

Defense system counts were analyzed via Negative Binomial GLMMs (fixed: Breed, Country; random: Collection) using *glmmTMB* and *DHARMa*. Pairwise comparisons were conducted via *emmeans* (Benjamini–Hochberg adjusted). We further regressed viral counts against DS/MAG ratios to evaluate their relationship, with significance defined as *P* < .05 and model fit by R^2^. Further details on the packages used are provided in the Supplementary Material (Supplementary Text 1).

## Results

### Global distribution of antiviral defense systems across cattle breeds and geographic origins

We have assembled a catalog to date of antiviral DS encoded in bovine rumen MAGs, totaling 90 205 DS across 6530 genomes (Supplementary Tables S1 and S2). The technical integrity of our dataset was confirmed by the inclusion of both genomic isolates and high-fidelity MAGs, all surpassing quality thresholds (≥ 50% completeness and ≤ 10% contamination) with a mean of 160 contigs per genome and an genome size 2.3 Mb, the majority exceeding the high-quality standards (>90% completeness and < 5% contamination) (Supplementary Fig. 1). On average, each genome encoded 22 DS, a pattern that was consistent across both bacterial and archaeal domains, with no difference observed between them. Although Archaea are typically less abundant in the rumen compared to bacteria (Supplementary Fig. 2), Nellore MAGs encoded the highest number of archaeal DS (*n* = 431), more than twice that observed in Luing (208), Charolais (189), Aberdeen Angus (139), Limousin (117), and Boran (43) (Supplementary Table S2).

**Figure 2:**
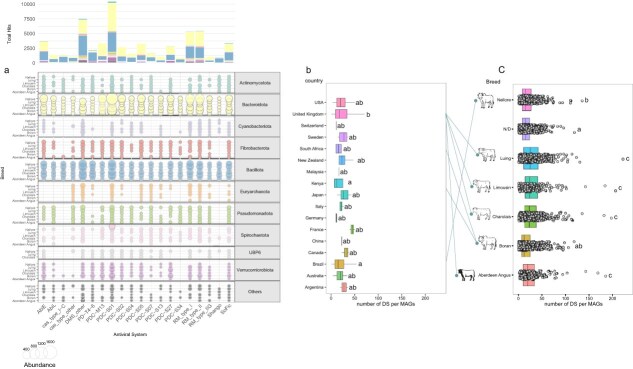
Comparative analysis of antiviral DS in rumen MAGs across cattle breeds and geographic origins. (a) Number of DS identified in MAGs grouped by cattle breed, including data from European, African, Brazilian cattle, and 405 genomes from the Hungate1000 catalogue. (b) Distribution of DS by country of origin and breed (c), encompassing both commercial taurine and Indian (zebu) lineages. Different letters denote statistically significant differences between groups (*P* > .05).

Our results highlight the existence of a broadly conserved core defensome, dominated by canonical systems, such as RM, deoxyribonucleic acid (DNA) modification systems (DMS_other), AbiE, and SoFic, along with recently identified phage defense candidates, including PDC-S01, S02, S05, and S27, all of which are widely distributed across cattle breeds (Fig. 2a). The RM system was the most abundant among the detected systems, with 10 739 annotations when types I, II, and IIG were combined. The PDC-S01 system was the most frequent individual *locus*, with 10 358 occurrences across all the breeds. Other highly abundant systems included AbiE (3734), SoFic (3357), and several additional PDC systems such as PDC-M13 (3324), PDC-S05 (3275), PDC-S27 (2865), PDC-S02 (2715), PD-T4-6 (2202), PDC-S07 (1672), and PDC-S04 (1628) (Supplementary Table S2). When stratified by breed, Nellore MAGs harbored the highest number of total antiviral systems (14851), followed by Boran (11206), Luing (10409), Charolais (9963), Aberdeen Angus (4287), and Limousin (3994) (Fig. 2a, Supplementary Table S2).

Antiviral DS exhibit a broadly distributed core across the bacterial families in the global dataset (Supplementary Fig. 3). Consistently, the most abundant and widely distributed bacterial family in the rumen harbored all major classes of antiviral DS, regardless of host breed (Supplementary Fig. 4a). Despite the presence of a shared core, the ruminal microbiome also exhibited breed-specific signatures, with certain genera uniquely associated with individual breed; UBA3824 and W3P20-009 (both Bacteroidota phylum), and bin RGB 10202 (Actinomycetota) were identified in Nellore cattle. Furthermore, CACZYB01 and CADAEX01 (both Pseudomonadota) were prevalent in the Luing breed, with the latter also observed in Limousin. Other associations included UBA11549 (Pseudomonadota) in Charolais, RFPC01 (Bdellovibrionota) in Boran, and the family *Xenobiaceae* in Aberdeen Angus. Regarding the system overlap across breeds, we found that 133 DS (including subtypes) were shared by all six cattle breeds (Supplementary Fig. 4b). Only a limited number of systems were exclusive to a single breed, and in most cases, they represent specific subtypes rather than entirely distinct families. For example, MAGs recovered from Charolais uniquely carried subtypes such as cas_type_I-A, AVAST_type_I, and brex_type_V, whereas those from Luing encoded argonaute_type_II/III and retron_XII. Exclusive subtypes in Nellore included druantia_type_III, and HEC-03 (Supplementary Fig. 4b). The genus-to-system-to-breed flow analysis revealed that despite quantitative variation in the abundance of antiviral DS across cattle breeds, a consistent set was shared among the major bacterial genera and breeds (Supplementary Fig. 4c).

When stratified by region, differences in the total DS abundance were observed (*P* < .05). The United Kingdom dataset of MAGs contained the largest number of DS (38478), followed by Africa (Kenya and South Africa) (11206), Brazil (14851), and New Zealand (4189) (Fig. 2b). Although this pattern is partly influenced by sampling depth (e.g. the UK dataset includes 4941 MAGs, whereas Brazil’s dataset comprises only 547 Nellore MAGs), it still contributed disproportionately, suggesting potential immune enrichment in this breed (Supplementary Fig. 5). Geographic hotspots of DS richness were evident in the UK, Brazil, and Kenya (Supplementary Fig. 6a).

The number of DS per MAG varied significantly among cattle breeds (Fig. 2b). Luing, Charolais, Aberdeen Angus, and Limousin exhibited the highest median DS counts, with no differences between them (groups c). Nellore showed intermediate values and overlapped partially with the upper group, while Boran MAGs had fewer DS on average, forming a distinct group (ab, *P* < .05). The composition of the antiviral DS varied across cattle breeds, although the most abundant systems were consistently shared (Supplementary Fig. 6b). This variation may also reflect differences in the distribution of Bacteria and Archaea across the breeds (Supplementary Fig. 7).

### Comprehensive analysis of the diversity of antiviral mechanisms in the ruminal microbiome of Nellore cattle

Despite having a smaller genomic representativeness, MAGs from the Nellore breed (Brazil) exhibited a disproportionately high arsenal of antiviral DS (Supplementary Fig. 5). We therefore conducted a more in-depth analysis of this dataset to investigate potential factors underlying this diversity. To our knowledge, no previous study has performed a detailed functional assessment of the DS in Nellore cattle.

**Figure 3:**
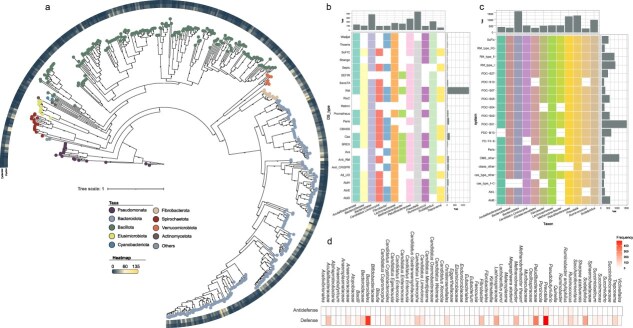
Diversity and distribution of antiviral DS in rumen MAGs from Nellore cattle. (a) Maximum-likelihood phylogenetic tree of 547 high-quality MAGs from the bovine rumen. The phylogeny was inferred using 120 single-copy protein-coding marker genes identified using GTDB-Tk. Colored circles in the tree tips represent the major bacteria, as indicated in the legend. Two concentric heatmap rings annotate the presence of DS: the inner arc corresponds to PADLOC predictions, and the outer arc corresponds to DefenseFinder results. Color intensity reflects the number of DS identified per genome. Clades were annotated using bootstrap support values. The tree was drawn to scale, with branch lengths proportional to the evolutionary distances. (b) bacterial defense profiles identified using DefenseFinder among the 12 taxonomic groups with the highest abundance of antiviral systems. The horizontal bars indicate the total number of systems detected per group, and the top vertical bars represent the number of MAGs analyzed within each taxonomic group. (c) Mapping of antiviral systems in the same taxonomic groups using PADLOC. The horizontal bars indicate the total number of systems detected per group, and the top vertical bars represent the number of MAGs analyzed within each taxonomic group. (d) Functional classification of systems identified by DefenseFinder based on the antiviral activity type. Note: taxa reflect the lowest informative taxonomic level reached per MAG to preserve ecological data from genomes with limited taxonomic resolution at deeper ranks.

The phylogeny of 547 high-quality MAGs revealed a diverse microbial community, with dominance of the phyla Bacillota and Bacteroidota, followed by representatives of Pseudomonadota, Actinomycetota, and Spirochaetota (Fig. 3a). Antiviral defense annotations mapped onto the tree were broadly distributed across taxonomic groups, with heatmaps indicating the number of systems detected by both PADLOC and DefenseFinder. Overall, PADLOC identified a higher number of defense *loci.* A total of 3831 DS were identified using Defense Finder, and a markedly higher number (15 632 systems) were detected using PADLOC (Supplementary Tables S3 and S4). The superior sensitivity of PADLOC is reflected in the number of predicted DS, and in its ability to identify novel defense-related genes classified as PCD, which highlights its potential to uncover previously uncharacterized antiviral mechanisms.

The most prevalent DS identified by both tools were RM systems, which were particularly abundant and further subclassified into types I, II, and IIG by Defense Finder and PADLOC (Fig. 3b and c). In addition to RM, Defense Finder identified several canonical and recently characterized DS, including Cas (CRISPR-associated proteins), Prometheus, Septu, and Shango systems. Conversely, PADLOC demonstrated an expanded detection range by identifying not only RM subclasses, but also a series of novel or underexplored systems, such as PDC-S01, a viperin family antiviral radical SAM protein, PDC-S07, and AbiE.

When comparing taxonomic profiles based on the last assigned taxonomic level for each MAG, *Prevotella* and Bacteroidales were the most abundant taxa associated with the DS. Although *Prevotella* belongs to the order Bacteroidales, we treated it separately because of the higher resolution available for some genomes. PADLOC uniquely detected Christensenellales among the 12 most enriched taxa. In addition, the DefenseFinder results showed that most DS were associated with defense *loci* rather than with anti-defense elements. Only a few taxa carried the predicted anti-defense compounds (Fig. 3d). Finally, we quantified the total number of defense-associated genes and HMMER domains identified using DefenseFinder, resulting in 7198 genes and 57 068 domains, reflecting the remarkable genetic diversity of DS strategies within the Nellore rumen microbiome (Supplementary Fig. 8, Supplementary Tables S5 and S6).

### Putative viral genomes and gene abundance across rumen taxa of Nellore

**Figure 4:**
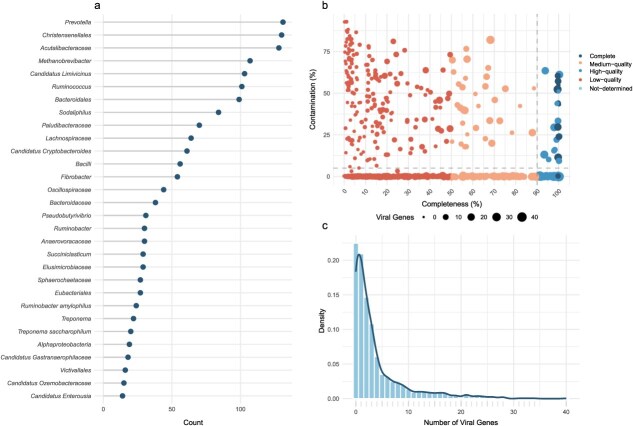
Detection and quality assessment of putative viral genomes within rumen MAGs. (a) Number of putative viral genomes identified by vSorter2 across different host-associated MAGs grouped by taxonomic affiliation. (b) Distribution of completeness and contamination levels of viral contigs assessed by CheckV. Dashed lines: High-quality thresholds (>90% Comp, <5% Cont). (c) Total number of viral genes detected within the MAGs of the most representative host taxa. Note: Taxa reflect the lowest informative taxonomic level reached per MAG to preserve ecological data from genomes with limited taxonomic resolution at deeper ranks.

Many putative viral genomes were detected across the 547 rumen MAGs, with certain genera, particularly *Prevotella, Ruminococcus*, and *Methanobrevibacter*, and the family *Lachnospiraceae* harboring higher counts, suggesting taxon-specific susceptibility to phage infection (Fig. 4a). A total of 1748 putative viral sequences were recovered, of which 1156 were classified as low-quality, 283 as medium to high quality, and 57 as provirus, indicating that both free and integrated viral forms were present within the host genomes (Fig. 4b). The number of viral genes varied among genera, with *Prevotella, Fibrobacter*, and the order Christensenellales displaying the highest gene counts, indicating a greater viral gene burden in these microbial lineages (Fig. 4c) (Supplementary Table S7).

To explore whether the presence of viral elements was associated with the abundance of antiviral DS, we performed a correlation analysis between the number of putative viral sequences per MAG and the total number of DS annotated in the corresponding host genome. Although the correlation was statistically significant (*R^2^* = 0.0712, *P* = 2.14 × 10^−7^), only 7.12% of the variation could be explained by the association between the number of viral elements and the diversity or abundance of antiviral defense mechanisms encoded in bacterial genomes (Supplementary Fig. 9). This indicates that additional factors likely contributed to the observed variations in the composition of the DS.

### Antiviral and anti-defense systems encoded in putative viral genomes from the microbiome of Nellore

**Figure 5:**
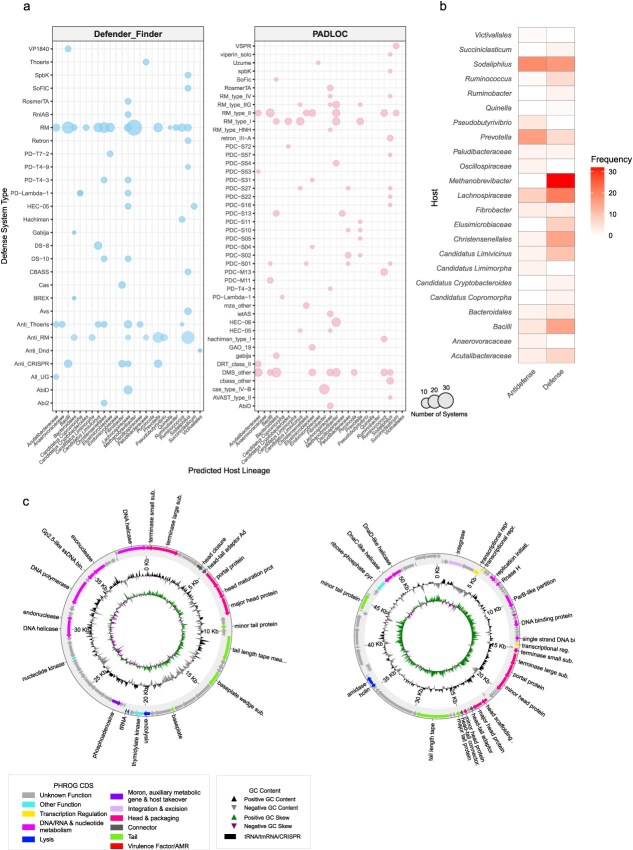
Antiviral DS encoded within putative viral genomes recovered from Nellore rumen MAGs. (a) Distribution of antiviral systems identified by PADLOC within putative viral genomes across 22 dominant host-associated taxa. (b) Functional classification of the detected antiviral systems based on predicted antiviral activity, performed with DefenseFinder. (c) Genome architecture of two representative viral contigs illustrating the co-localization of core viral genes, such as terminase, portal protein, major head protein, tail tape measure, and DNA polymerase, with predicted antiviral defense genes. Genome annotations also included regulatory, metabolic, and lysis-related gene functions. Note: taxa reflect the lowest informative taxonomic level reached per MAG to preserve ecological data from genomes with limited taxonomic resolution at deeper ranks.

A total of 384 antiviral DS were identified within putative viral sequences (vMAGs) detected in rumen MAGs, including 156 DS annotated by PADLOC and 228 annotated by DefenseFinder (Supplementary Tables S8 and S9). These DS were unevenly distributed among predicted viral hosts, with the highest counts observed in *Sodaliphilus* (*n* = 36), *Methanobrevibacter* (*n* = 32), *Lachnospiraceae* (*n* = 29), and *Prevotella* (*n* = 22), indicating these taxa may experience elevated viral pressure or harbor prophages carrying auxiliary defense elements (Fig. 5a).

Among the total DS mapped in the viral sequences, 155 were categorized as antiviral DS, with RM systems being the most prevalent (*n* = 95), followed by Abi, CBASS, SoFIC, and Retron elements. In contrast, 73 systems were classified as anti-DS, including anti-RM (*n* = 43) and anti-CRISPR (*n* = 13) systems (Fig. 5a and b). The relative enrichment of anti-DS in viral sequences, particularly the high number of anti-RM modules, highlights the evolutionary strategies employed by phages to evade host immunity, reinforcing the role of viruses not only as antagonists, but also as participants in the molecular arms race within microbial communities [22, 44].

Further structural and functional annotation of representative vMAGs revealed the coexistence of core viral genes (e.g. terminases, portal proteins, and tail fibers) and genes associated with auxiliary metabolic functions and antiviral DS, including tRNA genes, integration/excision modules, and defense island signatures (Fig. 5c).

### CRISPR spacer mapping highlights host–phage interactions in the rumen microbiota

**Figure 6:**
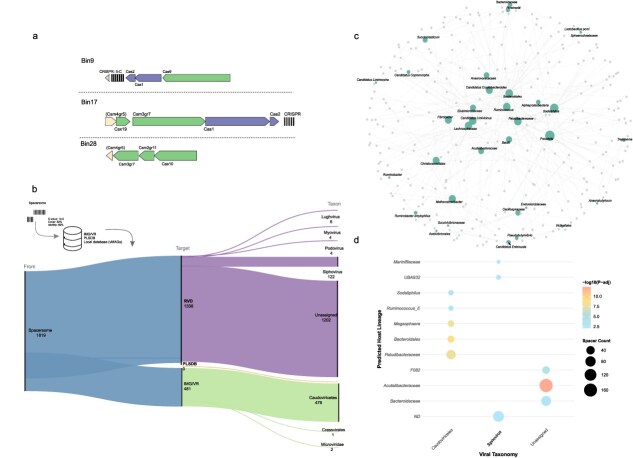
Characterization of CRISPR-Cas systems and viral targeting in ruminal MAGs. (a) Representation of the complete and incomplete CRISPR-Cas *loci* identified in selected MAGs, as previously described by Faleiros et al. (2024) [30]. (b) Overview of spacerome analysis from MAGs containing CRISPR-Cas systems, with spacer sequences compared with IMG/VR, PLSDB, RVD, and a custom local vMAGs database. (c) Virus–host interaction network in the rumen microbiome. Green nodes represent the identified host taxa (bacteria and archaea), while gray nodes represent the associated viral populations. Edges (lines) indicate predicted association relationships between viruses and hosts. The size of the viral nodes (gray) is scaled according to their numerical abundance, with larger circles indicating a higher count of viral sequences. (d) host–virus enrichment analysis based on CRISPR spacer-protospacer matches. The y-axis lists predicted host lineages, while the x-axis represents the statistical significance (−log_10_P-adj) of the associations. Bubble size indicates the total spacer count for each interaction. Note: taxa reflect the lowest informative taxonomic level reached per MAG to preserve ecological data from genomes with limited taxonomic resolution at deeper ranks.

To investigate the targeting activity of CRISPR-Cas systems encoded in the rumen MAGs, we performed a spacer match analysis using Spacersome, comparing CRISPR spacers against four reference datasets: IMG/VR, PLSDB plasmid, a local viral genome database (putative vMAGs), and the Virus database (RVD). Although the genomes were not fully closed, we identified and reconstructed CRISPR-Cas *loci* in some MAGs obtained from the rumen of Nellore cattle, allowing the characterization of gene synteny and effector diversity (Fig. 6a). These *loci* included conserved components, such as Cas1, Cas2, and Cas9, as well as type III-specific proteins, such as Csm3, Csm4, Cas10, and Csx19.

Spacer matching revealed 1819 significant hits, with the majority aligned to sequences in RVD (*n* = 1338), followed by IMG/VR (*n* = 481) (Supplementary Tables S10 and S11). No matches were observed in the PLSDB plasmid database, suggesting that CRISPR-Cas systems in rumen microbes primarily target viral rather than plasmid elements (Fig. 6b). When querying the local vMAG dataset, spacers matched viral contigs primarily associated with siphoviruses (*n* = 122) and the class Caudoviricetes (*n* = 478). To a lesser extent, matches were identified for myoviruses, podoviruses, and the genus *Lughvirus*, while a substantial portion (*n* = 1202) remained taxonomically unclassified.

The interaction network analysis revealed a complex architecture of associations between bacterial and archaeal hosts (represented in green) and their corresponding viral populations (represented in gray) (Fig. 6c). Among the primary host taxa identified, the phylum Bacteroidota was prominent, including MAGs belonging to the order *Bacteroidales* (the most specific informative taxonomic level reached) and the genera *Pre*votella and *Sodaliphilus*. Additionally, the genera *Ruminococcus* and *Fibrobacter* were highly represented, alongside key archaeal groups such as *Methanobrevibacter* (Fig. 6c). The network demonstrates that high-centrality hosts, particularly *Prevotella* and *Sodaliphilus*, are linked to multiple viruses of varying abundances, suggesting a diversified viral pressure on these dominant members of the microbiome.

The host–virus enrichment analysis revealed that most identified interactions are driven by members of the class Caudoviricetes (Fig. 6d). This viral group showed significant associations (P-adj < 0.05) across a wide range of host taxa, including *Sodaliphilus, Ruminococcus, Megasphaera, Bacteroidales*, and *Paludibacteraceae*. In these groups, the high Spacer Count suggests a robust and active history of viral exposure. In contrast, siphovirus phages were present, the host for most of these viral sequences could not be determined. Minor but specific associations were observed for *Marinifilaceae* and UBA932 (both Bacteroidota). Furthermore, a substantial portion of the viral signals was categorized as Unassigned (Fig. 6d).

### Transcriptional activity of antiviral Defense genes in rumen Metatranscriptomes

**Figure 7:**
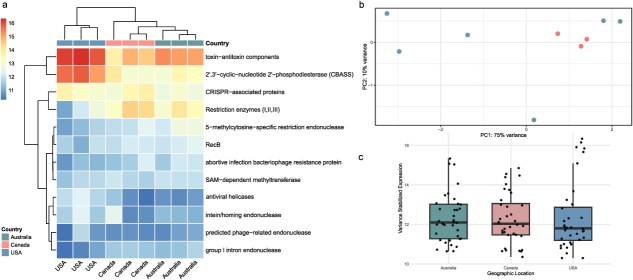
Transcriptional activity of antiviral defense genes in rumen metatranscriptomes from different geographic regions. (a) Hierarchical clustering and heatmap of normalized transcript abundance for key antiviral defense genes. Data were normalized using DESeq2 RLE, where the color scale represents relative expression levels (Z-score transformed), ranging from low (blue) to high (red) abundance. Samples are color-coded by geographic origin to highlight country-specific expression patterns. (b) PCA of the antiviral defensome metatranscriptomic profiles. The first two principal components, PC1 (75% variance) and PC2 (10% variance), demonstrate distinct clustering of microbial communities based on their geographic location. (c) Distribution of antiviral gene expression across geographic locations. Boxplots represent the VST of identified DS, showcasing the cumulative investment in microbial immunity within each country. Individual data points (jitter) represent the expression of specific gene categories.

Moreover, we analyzed publicly available rumen metatranscriptomic datasets to assess the transcriptional activity of genes involved in microbial antiviral defense. The transcriptional profile revealed that antiviral defense genes are actively expressed across all analyzed samples (Fig. 7). Although the primary objective was not a direct quantitative comparison between regions, the hierarchical clustering analysis demonstrated that samples naturally grouped by country of origin (USA, Canada, and Australia) (Fig. 7a). Specifically, high-standard DS exhibited the most robust transcriptional activity within the rumen metatranscriptomes. Systems such as toxin-antitoxin components, 2′,3′-cyclic-nucleotide 2′-phosphodiesterase (CBASS), CRISPR-associated proteins, and RM enzymes (Types I, II, and III) were identified as the most highly expressed defense categories. The Principal Component Analysis (PCA) further supports the differentiation in the transcriptional activity of microbial DS. The first two principal components explained 85% of the total variance (PC1: 75% and PC2: 10%), revealing a clear separation of samples based on their country of origin (Fig. 7b). Specifically, PC1 successfully segregated samples from the USA and Australia, while Canadian samples exhibited higher internal dispersion. However, despite these regional signatures, the results indicate a consistent level of transcriptional investment in antiviral mechanisms across Australia, Canada, and the USA (Fig. 7c). This is evidenced by the comparable distribution of variance-stabilized expression levels across the three geographic locations.

## Discussion

This study provides the most comprehensive mapping to date of antiviral DS within the rumen microbiome of *B. indicus* (Nellore and Boran) and *Bos taurus* (Charolais, Luing, Aberdeen Angus, and Limousin), expanding our understanding of the immune potential of this ecosystem and positioning it as a reservoir of both canonical and novel defense strategies. The rumen virosphere is diverse, harboring an extensive range of bacteriophages that shape microbial composition, nutrient turnover, and horizontal gene flow [18–20, 45]. Despite this complexity, studies focusing specifically on the viral components of the ruminal microbiome, particularly in cattle from diverse geographical regions, remain limited. We annotated DS using PadLoc-DB and DefenseFinder, expanding upon Saenz et al. [27], relied exclusively on PadLoc-DB. Our study includes 547 Nellore MAGs across diverse breeds and geographies. By integrating rumen transcriptomes, we validated the activity of these systems, identifying functionally relevant defense repertoires actively expressed within the microbiome.

On a global scale, our comparative analysis across breeds and countries revealed that both host lineage and geography significantly influence the distribution of DS. This variation mirrors patterns in rumen bacterial community composition, including the presence of genera unique to each breed, and is likely shaped by factors such as diet, species, sex, and breed [46, 47]. Although the dataset size partially explains the high system counts in the UK and Kenyan MAGs, the notably high immune potential in Nellore cattle based on a smaller dataset suggests an enriched or compact immune repertoire. The interpretation of these results requires a thorough analysis, given the interaction between biological factors including the diet provided to the animals, variations in the composition and interactions of the microbiome, and gene expression in the host animal [48], in addition, it is known that the host’s genetics affects the composition of the rumen microbial communities [11, 49]. The abundance of DS was also more similar among breeds within the same genetic group (zebu vs. taurine) (Fig. 2a and c), which reflects the influence of genetic factors and possibly reflecting shared physiological traits such as rumen volume and feeding behavior that affect microbiome structure [50]. Additionally, breed-specific differences may result from unique phage exposure histories and host-selective pressures that influence virome dynamics [19].

Considering the ruminal ecosystem, the mapping carried out in this study revealed that, on average, each genome encoded 22 DS, a density that far exceeds that reported for other microbiomes, such as soil and the human gut [51]. This includes a high abundance of well-known DS, such as RM and AbiE, which were also prevalent in a previous study [27]. However, our analysis also uncovered a high abundance of single- and multi-gene PDC systems, which are typically poorly associated with other defense *loci* and remain underexplored in ruminal environments [52]. According to a recent study [53], PDC systems are enriched in Archaea, with PDC-S01 and PDC-S27 being among the most abundant DS. PDC-S27 originated from Archaea and subsequently spread to bacteria. These patterns align with the distribution of breeds and possibly diets, with Nellore, Luing, and Charolais cattle showing a higher abundance of Archaea and the PDC-S01 defense system. Furthermore, a recent large-scale study on aquifer microbiomes mapped 90 824 DS across 29 031 MAGs, demonstrating the widespread occurrence of antiviral systems in aquatic environments [54]. Although this dataset was approximately 4.5 times larger than ours, the total number of DS identified was comparable to that observed in the rumen.

Nellore cattle, which are widely used in Brazilian beef production and are well adapted to low-input environments [55], represent an ecologically and economically important model for studying host breed–microbe–virus interactions. RM systems dominate the Nellore rumen antiviral landscape, alongside CRISPR, AbiE, and SoFIC. This diversity suggests a redundant, layered immune architecture against intense viral predation, consistent with distributed immunity theories in complex microbial communities [24].

Antiviral DS were predominantly encoded by genus *Prevotella* and the order Bacteroidales, reflecting their ecological dominance in the rumen microbiome, as extensively documented across host species, geographic locations, and ruminant lineages [14, 46, 56–59]. *Prevotella spp*. thrive in the anaerobic, fiber-rich environment of the rumen, playing key roles in protein metabolism, hemicellulose degradation, and volatile fatty acid production, which directly contribute to host nutrition [60, 61]. Members of the order Bacteroidales are similarly prevalent and functionally relevant in this context [62]. As metabolically dominant taxa, Prevotella and Bacteroidales likely face high viral predation, driving DS enrichment. Supporting this, these taxa harbor the highest counts of DS, prophages, and viral genes, reflecting intense and historical virus–host interactions.

The detection of 1748 putative viral genomes enabled the exploration of the virus–host arm race in rumen MAGs. Although modest, the positive correlation between viral sequence count and antiviral defense system abundance suggests that phage pressure contributes to immune repertoire diversification, consistent with patterns observed in other ecosystems [51]. Among the hosts, Christensenellales stood out as the taxon with the highest number of putative viral genomes and genes, yet it exhibited a notably low representation of anti-DS. This order was among the top in PADLOC, particularly enriched for novel defense *loci,* but was absent from the DefenceFinder top hits. As a member of the phylum Bacillota and class Clostridia, Christensenellales is part of one of the most abundant phyla in the rumen exhibiting defensive systems [27]. A reduced antiviral repertoire appears to directly contribute to elevated phage prevalence in Christensenellales, highlighting the need for fine-scale taxonomic analysis.

The detection of 384 defense and 73 anti-DS (e.g. anti-CRISPR, anti-RM, anti-Thoeris) within vMAGs reveals a sophisticated viral counter-defense repertoire. This highlights an intense co-evolutionary arms race, where rumen phages actively manipulate or bypass host immunity to ensure persistence. These systems enable phages to subvert key components of the bacterial immune arsenal [63]. Anti-CRISPR proteins inhibit CRISPR-Cas activity by directly blocking Cas effectors or interfering with spacer acquisition, thereby neutralizing adaptive immunity [64]. Anti-RM systems protect phage genomes from degradation by host restriction–modification complexes, often through methylation mimicry or the inhibition of restriction enzymes [65]. Anti-Thoeris elements suppress the recently described Thoeris defense pathway, which relies on NAD^+^-based signaling to trigger cell suicide upon infection [66]. Taken together, our data underscore the dual role of viruses as targets and immune function vectors.

Finally, the active expression of CRISPR, restriction, and toxin-antitoxin systems under non-infectious conditions points to a basal state of immunological readiness. This suggests that rumen microbes maintain constitutive defenses to withstand persistent pressure from the diverse local virome [67]. The predominance of toxin-antitoxin modules observed in this study may be associated with nutrient availability in the environment. For example, RNase-type toxins, such as YafQ and RelE, showed elevated transcription levels under fatty acid deprivation, indicating activation in the face of metabolic stress [68]. This implies that dietary composition and availability could play a role in modulating disruptions in ruminal homeostasis [69]. Therefore, the presence of possibly stressful conditions in complex environments such as the rumen can induce adaptive responses of DS in bacterial communities [70], furthermore, a greater prevalence of DS is observed in more challenging environments [51].

In conclusion, this study demonstrates that the architecture of antiviral DS in the rumen is a direct reflection of host phylogeny and geography. The clear distinction between Zebu (e.g. Nellore) and Taurine breeds in DS distribution reveals that host genetics exert selective pressure on the microbial immune arsenal. We identified that *Prevotella* and Bacteroidales MAGs function as hotspots for antiviral defenses and prophage elements, while vMAGs carry a sophisticated counter-offensive of anti-defense genes (anti-CRISPR, anti-RM). Crucially, our metatranscriptomic data validate that these systems are not only present but are actively expressed within the rumen environment.

## Supplementary Material

Supplementary_Material_ycag162

## Data Availability

All data supporting the findings of this study are available in the article and Supplementary Materials. Genome assemblies and raw data for the Nellore rumen MAGs were retrieved from two NCBI BioProjects. Faleiros et al. (2024) [30] PRJNA1054691; Conteville et al. (2024) [31]: PRJNA98774335. The comparative genome dataset was obtained from the Cow Rumen Genome Catalogue v1.0, accessible via MGnify at https://www.ebi.ac.uk/metagenomics/rumen. This resource includes 4941 MAGs generated from rumen metagenomic datasets across multiple countries (Stewart et al., 2019) [28], 1200 MAGs from African cattle, as published by Wilkinson et al. (2020) [29], and 410 reference genomes from the Hungate1000 Collection (Seshadri et al., 2018) [5].
