# Supplementary material for: Host breed and geography shape the antiviral defense landscape of the bovine rumen microbiome
Source: ISME Commun. 2026 Jun 11;6(1):ycag162. doi: 10.1093/ismeco/ycag162 (PMC13356809; doi:10.1093/ismeco/ycag162)
Supplement: Supplementary_Material_ycag162 [file supplementary_material_ycag162.zip › Supplementary_Figures_ycag162.docx]

**Host Breed and Geography Shape the Antiviral Defense Landscape of the Bovine Rumen Microbiome**

Camila A. Faleiros^1#^, Osiel S. Gonçalves^1,2#*^, Alanne T. Nunes^1^, Crislaine S. Pires^1^, Mirele D. Poleti^1^, Heidge Fukumasu^1*^

^1^Department of Veterinary Medicine, School of Animal Science and Food Engineering (FZEA), University of São Paulo (USP), Avenida Duque de Caxias Norte, 225 - Jardim Elite, Pirassununga, SP (ZIP Code 13635-900), Brazil.

^2^Department of Biological Science, Microbial Eco-Evolutionary Genomics Group, Midwestern Parana State University (Unicentro), Campus CEDETEG, Alameda Élio Antonio Dalla Vecchia, 838 - Vila Carli (85040-167), Guarapuava, PR (ZIP Code 85040-167), Brazil.

^#^These authors contributed equally to this work

Correspondence:

[osiel.goncalves@unicentro.br](mailto:osiel.goncalves@unicentro.br) (O. Gonçalves, 0000-0002-6311-799X)
[fukumasu@usp.br](mailto:fukumasu@usp.br) (H. Fukumasu, 0000-0002-3265-5090)

**Supplementary Figures**

**Supplementary Figure 1** - Quality assessment and assembly metrics of genomic isolates and MAGs across different cattle breeds.

**A**, Raincloud plot displaying the distribution of total genome length (bp), showing consistent assembly sizes among the studied groups. **B**, Distribution of the number of contigs per assembly, illustrating the fragmentation level across different breeds. **C**, Scatter plot of completeness versus contamination percentages. Points are colored by host breed and sized by N50 values. The red dashed lines indicate the high-quality thresholds (completeness >90% and contamination <5%).

**Supplementary Figure 2** – Domain-Level Abundance of Defense Systems (DS) in Bovine Rumen Metagenomes


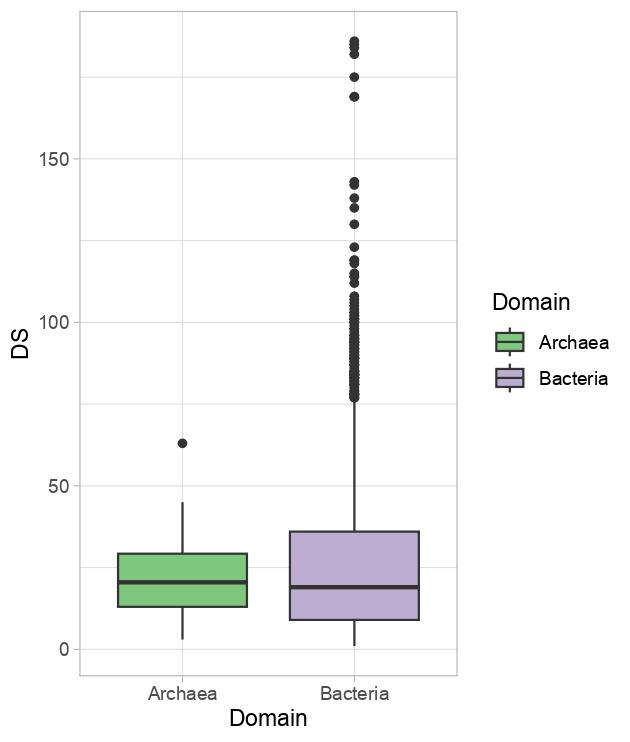


Abundance of defense systems across domains in bovine rumen metagenomes. Boxplot showing the distribution of the number of defense systems detected in archaeal and bacterial metagenome-assembled genomes (MAGs).

**Supplementary Figure 3** – Distribution of the 20 most representative antiviral systems across the 10 most abundant bacterial families in the global dataset.


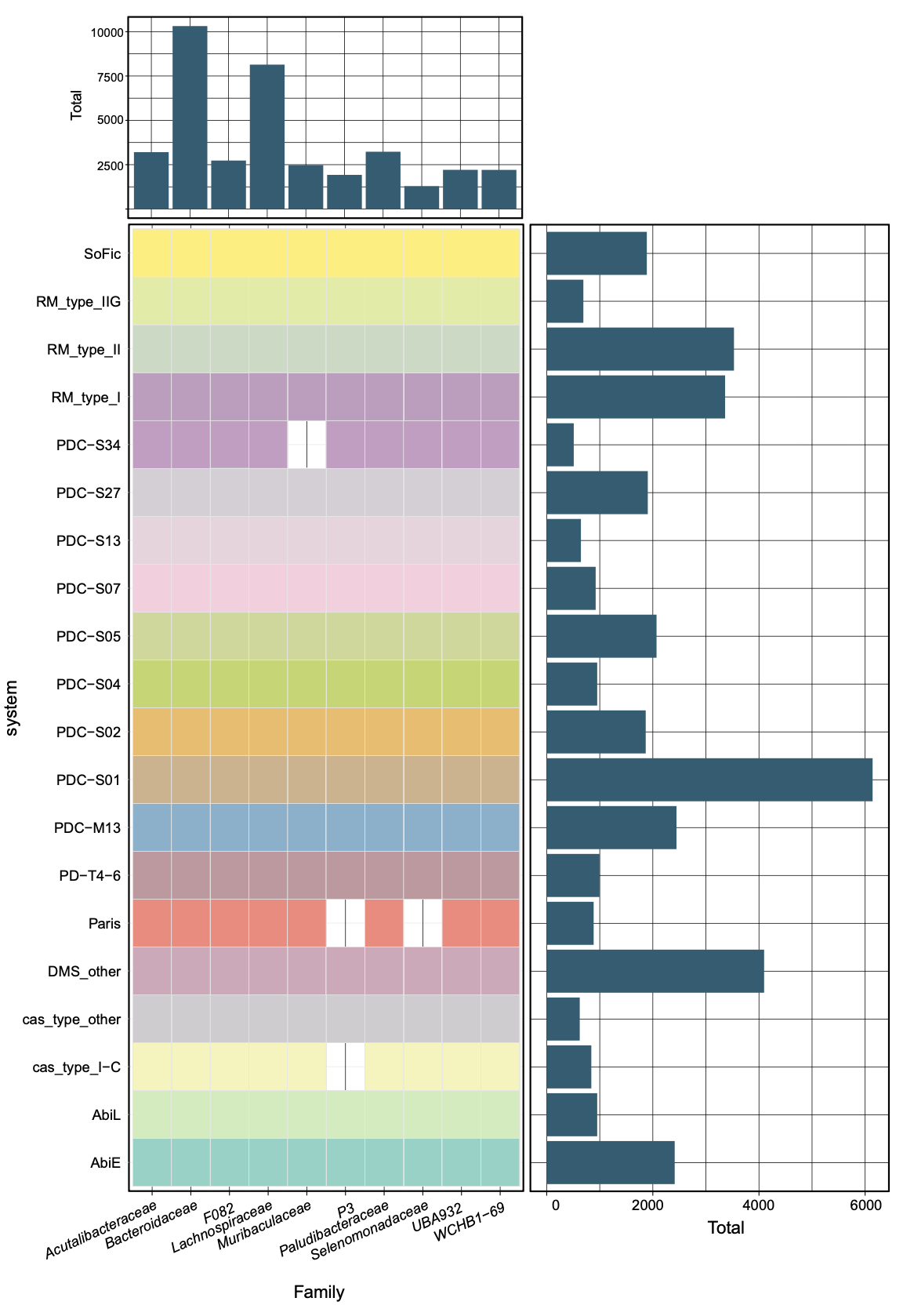


The central heatmap (geom_tile) displays the presence of each antiviral system (rows) within each bacterial family (columns), with colored tiles indicating system occurrence and white tiles indicating absence. The bar plot on the right shows the total number of hits for each antiviral system, while the top bar plot shows the total number of antiviral systems detected per family.

**Supplementary Figure 4** – Detailed Analysis of the Abundance of Antiviral Defense Systems by Cattle Breed


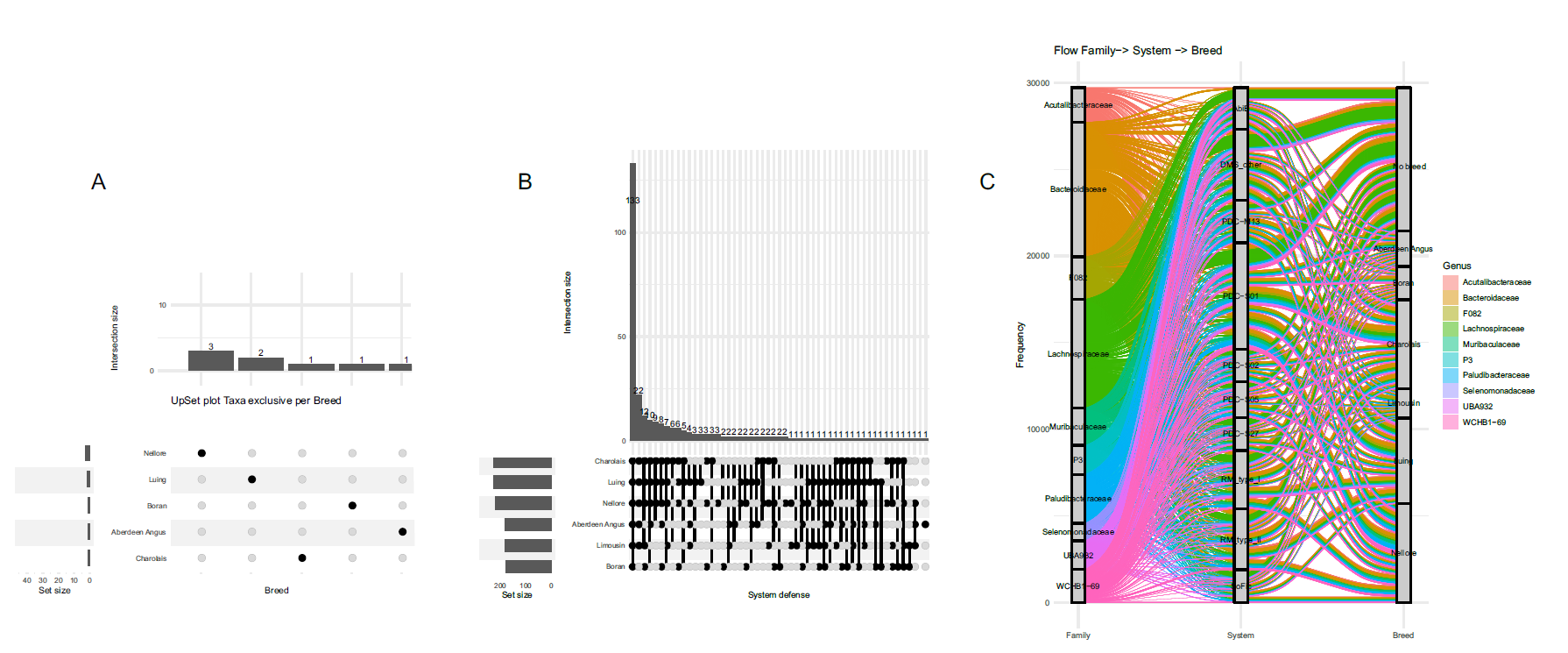


**A**, Number of unique rumen microbial taxa identified for each cattle breed. **B**, UpSet plot showing the intersections of antiviral defense systems detected across six cattle breeds. Vertical bars at the top represent the number of defense systems shared among breeds, as indicated by the connected dots below. This plot highlights both conserved sets of microbial defense systems and those restricted to specific subsets of breeds or unique to a single breed (columns with a single dot). Horizontal bars on the left indicate the total number of defense systems detected in each breed. **C**, Relationship between microbial taxonomic groups, types of antiviral defense systems, and cattle breeds. The width of each flow reflects the abundance of taxa and associated defense systems within the respective cattle breeds.

**Supplementary Figure 5 -** Correlation between the total number of Metagenome-Assembled Genomes (MAGs) and the total number of identified Defense Systems (DS) per breed.

The size of each bubble represents the DS per MAG ratio (calculated as the total number of DS divided by the total number of MAGs per dataset)

**Supplementary Figure 6 - Comparative analysis of antiviral defense systems in rumen MAGs across cattle breeds and geographic origins.**

**A**, Heatmap displaying the number of defense systems detected per country, highlighting geographic trends in the antiviral repertoire of the rumen microbiome. **B**, the ten most prevalent antiviral defense systems in the rumen, organized by cattle breeds.

**Supplementary Figure 7** – Abundance and Distribution of Bacterial and Archaeal Domains Among Cattle Breeds


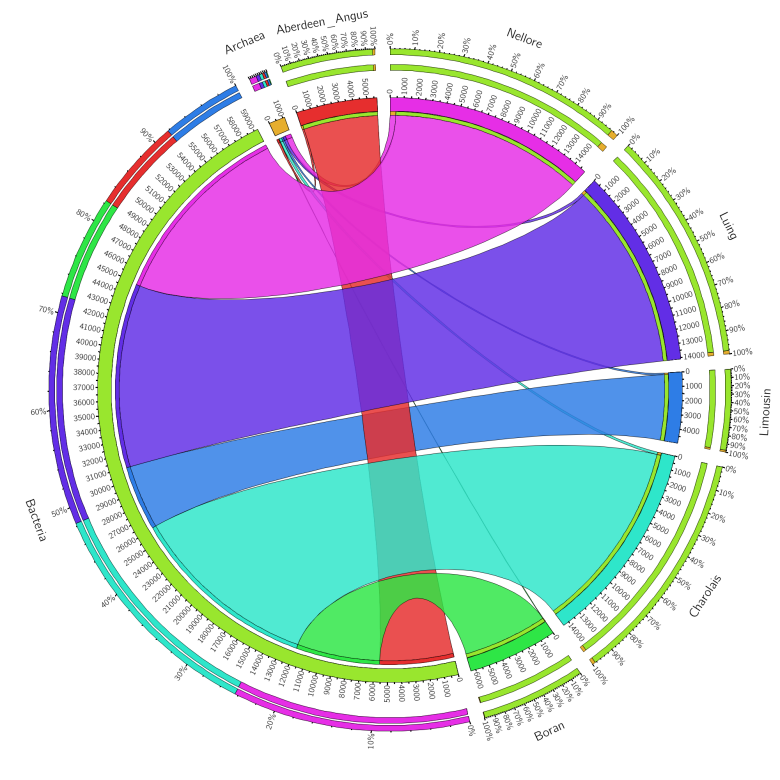


Diagram representing the connection between microbial domains (Bacteria and Archaea) and different cattle breeds based on the abundance of taxa identified in rumen metagenomes. The outer arcs indicate the relative contribution of each domain and breed to the total number of observed taxa. The colored inner ribbons connect microbial domains to cattle breeds, reflecting the taxonomic origin of microorganisms associated with each group. The width of each connection is proportional to the abundance of taxa linked to each domain within each breed.

**Supplementary Figure 8** – Annotation of Genes and HMM Domains Associated with Antiviral Defense Systems Using DefenderFinder in Rumen Microbial Genomes from Nellore Cattle


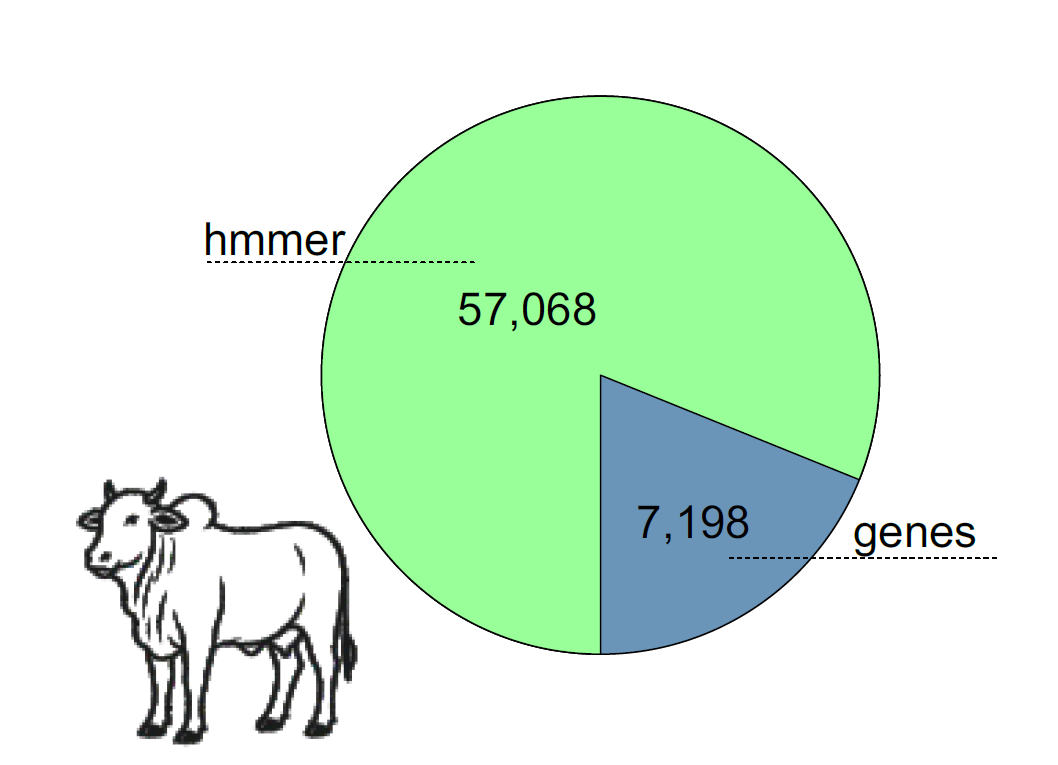


Overall quantification of defense-related genes and HMMER domains as identified by DefenseFinder.

**Supplementary Figure 9** – Correlation Between Predicted Viral Sequences and Antiviral Defense System Abundance in Microbial Genomes


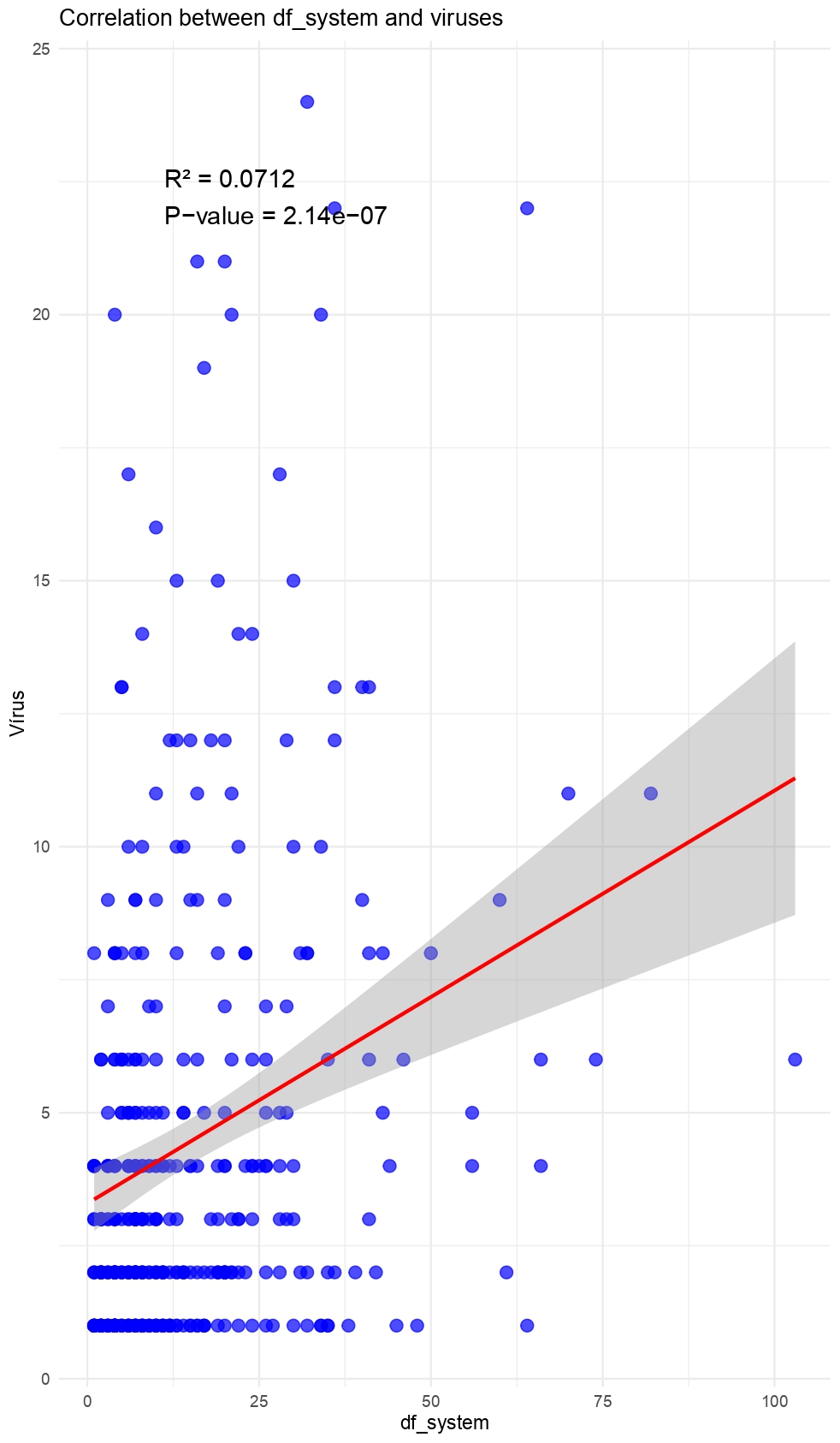


Scatterplot showing the correlation between the number of predicted viral sequences and the abundance of antiviral defense systems in individual rumen microbial genomes. Each blue dot represents a single microbial genome, with the x-axis indicating the number of defense systems and the y-axis representing the number of viral sequences associated with that genome. The red line represents the linear regression fit, with the shaded area indicating the 95% confidence interval.
