# Supplementary material for: Host breed and geography shape the antiviral defense landscape of the bovine rumen microbiome
Source: ISME Commun. 2026 Jun 11;6(1):ycag162. doi: 10.1093/ismeco/ycag162 (PMC13356809; doi:10.1093/ismeco/ycag162)
Supplement: Supplementary_Material_ycag162 [file supplementary_material_ycag162.zip › Supplementary_Text_1_ycag162.docx]

**Host Breed and Geography Shape the Antiviral Defense Landscape of the Bovine Rumen Microbiome**

Camila A. Faleiros^1#^, Osiel S. Gonçalves^1,2#*^, Alanne T. Nunes^1^, Crislaine S. Pires^1^, Mirele D. Poleti^1^, Heidge Fukumasu^1*^

^1^Department of Veterinary Medicine, School of Animal Science and Food Engineering (FZEA), University of São Paulo (USP), Avenida Duque de Caxias Norte, 225 - Jardim Elite, Pirassununga, SP (ZIP Code 13635-900), Brazil.

^2^Department of Biological Science, Microbial Eco-Evolutionary Genomics Group, Midwestern Parana State University (Unicentro), Campus CEDETEG, Alameda Élio Antonio Dalla Vecchia, 838 - Vila Carli (85040-167), Guarapuava, PR (ZIP Code 85040-167), Brazil.

^#^These authors contributed equally to this work

Correspondence:

[osiel.goncalves@unicentro.br](mailto:osiel.goncalves@unicentro.br) (O. Gonçalves, 0000-0002-6311-799X)
[fukumasu@usp.br](mailto:fukumasu@usp.br) (H. Fukumasu, 0000-0002-3265-5090)

**Supplementary Text**

**Materials and Methods**

**Comparative Dataset Construction of Bovine Rumen Microbial Genomes**

To establish a comprehensive genomic framework for exploring antiviral defense systems in the bovine rumen, we assembled a curated dataset of 5,983 high-quality microbial genomes and metagenome-assembled genomes derived from rumen samples across multiple cattle breeds and geographic regions. This included three major sources: (i) the Cow Rumen Genome Catalogue v1.0, accessible via MGnify (https://www.ebi.ac.uk/metagenomics/ rumen), comprising 5,174 MAGs generated from 60 independent rumen metagenomic datasets[1]; (ii) 404 MAGs from African cattle [2]; and (iii) 405 reference genomes of isolates from the Hungate1000 Collection[3]. Only genomes explicitly annotated as rumen-derived were retained. Metadata on cattle breed, geographic origin, and study descriptors were extracted from the original publications for downstream comparative analyses (Supplementary Table S1).

As a focal subset, we included 547 high-quality MAGs from Nellore cattle (B. indicus), raised in tropical regions of Brazil. The genomes were retrieved from two publicly available studies[4,5] (NCBI BioProject PRJNA1054691 and PRJNA98774335). The dataset comprised high-quality genomes previously filtered based on CheckM[6] metrics, strictly adhering to thresholds of ≥80% completeness and ≤10% contamination, with a final quality score ≥50 (Supplementary Table S1). Taxonomic classification of all MAGs, both global and Nellore subsets, was performed using the GTDB-Tk toolkit (v2.4.0) against the Genome Taxonomy Database (GTDB) release R220[7]. This unified and metadata-informed genome collection encompassed 6,530 rumen-derived MAGs. Notably, the taxa reported reflect the lowest informative taxonomic level reached per MAG. This approach was adopted to preserve crucial ecological data from genomes that exhibited limited taxonomic resolution at deeper ranks (e.g., genus or species).

**Annotation and Identification of Antiviral Defense Systems in Rumen MAGs**

To characterize antiviral defense in the bovine rumen microbiome, we analyzed 6,530 high-quality MAGs and isolates genomes. Annotation was performed using Prokka v1.14.6[8], generating protein sequence files (*.faa) used as input for downstream defense system prediction.

We employed PADLOC v2.0.0 (Prokaryotic Antiviral Defence LOCator)[9] to identify canonical and novel antiviral systems. PADLOC uses Hidden Markov Models (HMMs) to screen prokaryotic genomes against a curated catalog of experimentally validated and candidate defense loci. This enabled the detection of a broad range of antiviral mechanisms, including well-characterized systems as well as recently described or underexplored systems, such as phage defense candidate (PCD) loci. To validate the predictions, all the detected systems were cross-checked against the PADLOC-DB reference database.

**Annotation and Identification of Antiviral Defense Systems in Nellore MAGs**

We analyzed 547 high-quality MAGs to characterize the antiviral defense repertoire encoded in the rumen microbiome of Nellore cattle. Protein sequence files (*.faa) generated during genome annotation were used as inputs for defense system detection using two complementary tools. We first applied PADLOC v2.0.0[9], followed by DefenseFinder v2.0.0[10], with the --antidefensefinder option enabled to allow simultaneous detection of antiviral systems and viral-encoded anti-defense elements. To validate and contextualize all predictions, the detected systems were cross-checked against the PADLOC-DB reference database and DefenseFinder Wiki (<https://defense-finder.mdmparis-saclay.fr/>). Phylogenomic classification was performed using GTDB-Tk v2.4.0 (GTDB R207)[7] based on 120 bacterial and 53 archaeal conserved marker genes. The reconstruction was performed using IQ-TREE v1.6.11[11], applying automatic model selection and ultrafast bootstrap analysis (‘-m TEST -bb 1000′). The resulting maximum-likelihood phylogenetic tree was visualized using the Interactive Tree of Life (iTOL) platform[12].

**Detection of putative viral elements and identification of antiviral systems in rumen MAGs from Nellore**

The objective of this analysis was to detect and quantify putative viral sequences embedded within bacterial MAGs as a first step toward investigating the relationship between viral load and host-encoded antiviral defense systems. We analyzed 547 high-quality MAGs derived from Nellore rumen, which were screened using VirSorter2 (v2.2.3) with the parameters *--min-length 1500 and --min-score 0.8* to prioritize high-confidence viral predictions[13]. Only sequences containing ≤ 2 viral hallmark genes were retained, reducing the likelihood of false positives. Quality and completeness were evaluated with CheckV (v1.0.1)[14]. Curation followed a stringent protocol based on the VirSorter2 SOP. Contigs were retained if they contained viral hallmark genes, had high prediction scores (≥0.95), or no detectable host or viral genes. Contigs dominated by host genes or those with insufficient viral signals were discarded, whereas ambiguous cases (e.g., those with a single host gene and ≥10 kb in length) were manually reviewed. The full description of this topic is in the supplementary material (Supplementary Text).

To investigate the functional potential of these viral elements, all curated viral contigs were screened for immune-related genes using PADLOC and DefenseFinder, with the *--antidefensefinder* flag enabled. In addition, the viral-host interaction landscape was characterized through two complementary approaches. First, a host-provirus interaction network was constructed from a presence-absence matrix of proviruses and their respective bacterial hosts. Network architecture, topological properties, and visualization were performed using the *igraph* package in R (version 4.4.1).

**Detection and Characterization of CRISPR-Cas Systems and Spacer Matching Against Viral and Plasmid Databases**

To identify CRISPR-Cas systems within the rumen microbial genomes previously assembled and annotated as containing antiviral Cas genes, we employed CRISPRCasTyper[15], which detects CRISPR arrays and Cas gene clusters as well as the prediction of CRISPR-Cas system subtypes based on the combination of Cas genes and the repeat sequences of CRISPR arrays. Only the genomes confirmed to harbor Cas antiviral systems were included in the analysis. Following CRISPR-Cas detection, all spacer sequences identified within these CRISPR arrays were extracted to compose a query dataset called Spacersome. The Spacersome dataset was used to assess the viral targets potentially recognized by rumen microbial CRISPR systems.

To determine the potential viral and plasmid origins of the spacers, we conducted similarity searches against four comprehensive reference datasets using blastn in a local environment: (1) the IMG/VR viral spacer BLAST database, a curated nucleotide viral database[16], with an e-value cutoff of 1e-5 for significant matches; (2) PLSDB plasmid database (version 2024_05_31_v2)[17], comprising a broad collection of plasmid sequences; (3) Local viral genome database constructed from putative vMAGs obtained from rumen metagenomic assemblies; and (4) Rumen Virome Database (RVD)[18], a specialized database representing the rumen viral community. Significant spacer matches were defined as those with e-values of ≤ 1e-5. Viral taxonomic information was retrieved from IMG/VR and RVD metadata.

A statistical enrichment analysis was conducted based on CRISPR spacer-protospacer matches. Viral taxonomy and predicted host lineages were retrieved from the RVD and the IMG/VR database. For each host-virus pair, enrichment was assessed using Fisher’s Exact Test (P<0.05), with P-values adjusted via the Benjamini-Hochberg (BH) method to control the false discovery rate.

**Metatranscriptomic Analysis**

To assess antiviral gene expression, metatranscriptomic datasets were retrieved from the JGI IMG/M portal (https://img.jgi.doe.gov). The study included three distinct geographic groups: (i) three rumen fluid metatranscriptomes from dairy cows at UC Davis, California, USA (Study IDs: 3300036544, 3300036316, and 3300036545); (ii) three Metatranscriptomes of bovine rumen microbial communities from Lethbridge, Alberta, Canada (Study IDs: 3300039431, 3300037691, and 3300037698); and (iii) three Metatranscriptomes of bovine rumen microbial communities from tropical cattle in Woodstock, Queensland, Australia (Study IDs: 3300042986, 3300037685, and 3300037684).

RNASeq expression data (raw read counts) were downloaded for all samples. Genes were filtered based on their functional annotation related to the antiviral defensome. Downstream statistical analyses were performed in R. To address the compositional nature of metatranscriptomic data and account for differences in library size between geographic locations, raw counts were normalized using the DESeq2 package. Specifically, the Relative Log Expression (RLE) method was employed to estimate size factors and calculate normalized abundances.

To stabilize variance across the range of expression levels, a Variance Stabilizing Transformation (VST) was applied using the *varianceStabilizingTransformation* function, which is particularly robust for datasets with a focused number of genes.

**Statistical analysis and visualization**

To compare defense systems counts among cattle breeds and countries of origin, we employed a Generalized Linear Mixed Model (GLMM) with a Negative Binomial distribution (*nbinom2*) to account for the overdispersion typical of genomic count data. The model included Animal Breed and Country as fixed effects, while Collection (source dataset) was incorporated as a random effect to account for the nested structure of the metagenomic data. All analyses were performed in R using the *glmmTMB* package for model fitting. Model residuals were validated for consistency using the *DHARMa* package. Post-hoc pairwise comparisons were conducted using Estimated Marginal Means via the *emmeans* package, with P-values adjusted by the Benjamini-Hochberg (BH) method. To investigate the relationship between viral content and antiviral defense system abundance in rumen MAGs, we applied a linear regression model using R. The model was fitted with the number of predicted viral sequences as the dependent variable, and the total number of defense systems per MAG as the independent variable. Model performance was assessed using the coefficient of determination (R²), and statistical significance was determined using the p-value (p < 0.05) associated with the regression slope.

**Reference**

1. Stewart RD, Auffret MD, Warr A *et al.* Compendium of 4,941 rumen metagenome-assembled genomes for rumen microbiome biology and enzyme discovery. *Nature Biotechnology 2019 37:8* 2019;**37**(8):953–61. https://doi.org/10.1038/s41587-019-0202-3.

2. Wilkinson T, Korir D, Ogugo M *et al.* 1200 high-quality metagenome-assembled genomes from the rumen of African cattle and their relevance in the context of sub-optimal feeding. *Genome Biol* 2020;**21**(1):1–25. https://doi.org/10.1186/S13059-020-02144-7/TABLES/7.

3. Seshadri R, Leahy SC, Attwood GT *et al.* Cultivation and sequencing of rumen microbiome members from the Hungate1000 Collection. *Nature Biotechnology 2018 36:4* 2018;**36**(4):359–67. https://doi.org/10.1038/nbt.4110.

4. Faleiros CA, Nunes AT, Gonçalves OS *et al.* Exploration of mobile genetic elements in the ruminal microbiome of Nellore cattle. *Scientific Reports 2024 14:1* 2024;**14**(1):1–12. https://doi.org/10.1038/s41598-024-63951-7.

5. Conteville LC, Silva JV da, Andrade BGN *et al.* Recovery of metagenome-assembled genomes from the rumen and fecal microbiomes of Bos indicus beef cattle. *Sci Data* 2024;**11**(1):1385. https://doi.org/10.1038/s41597-024-04271-3.

6. Parks DH, Imelfort M, Skennerton CT *et al.* CheckM: assessing the quality of microbial genomes recovered from isolates, single cells, and metagenomes. *Genome Res* 2015;**25**(7):1043. https://doi.org/10.1101/GR.186072.114.

7. Chaumeil PA, Mussig AJ, Hugenholtz P *et al.* GTDB-Tk v2: memory friendly classification with the genome taxonomy database. *Bioinformatics* 2022;**38**(23):5315–6. https://doi.org/10.1093/bioinformatics/btac672.

8. Seemann T. Prokka: rapid prokaryotic genome annotation. *Bioinformatics* 2014;**30**(14):2068–9. https://doi.org/10.1093/BIOINFORMATICS/BTU153.

9. Payne LJ, Meaden S, Mestre MR *et al.* PADLOC: a web server for the identification of antiviral defence systems in microbial genomes. *Nucleic Acids Res* 2022;**50**(W1):W541–50. https://doi.org/10.1093/NAR/GKAC400.

10. Tesson F, Hervé A, Mordret E *et al.* Systematic and quantitative view of the antiviral arsenal of prokaryotes. *Nature Communications 2022 13:1* 2022;**13**(1):1–10. https://doi.org/10.1038/s41467-022-30269-9.

11. Minh BQ, Schmidt HA, Chernomor O *et al.* IQ-TREE 2: New Models and Efficient Methods for Phylogenetic Inference in the Genomic Era. *Mol Biol Evol* 2020;**37**(5):1530–4. https://doi.org/10.1093/molbev/msaa015.

12. Letunic I, Bork P. Interactive Tree of Life (iTOL) v6: recent updates to the phylogenetic tree display and annotation tool. *Nucleic Acids Res* 2024;**52**(W1):W78–82. https://doi.org/10.1093/nar/gkae268.

13. Guo J, Bolduc B, Zayed AA *et al.* VirSorter2: a multi-classifier, expert-guided approach to detect diverse DNA and RNA viruses. *Microbiome* 2021;**9**(1):1–13. https://doi.org/10.1186/S40168-020-00990-Y/FIGURES/5.

14. Nayfach S, Camargo AP, Schulz F *et al.* CheckV assesses the quality and completeness of metagenome-assembled viral genomes. *Nature Biotechnology 2020 39:5* 2020;**39**(5):578–85. https://doi.org/10.1038/s41587-020-00774-7.

15. Russel J, Pinilla-Redondo R, Mayo-Muñoz D *et al.* CRISPRCasTyper: Automated Identification, Annotation, and Classification of CRISPR-Cas Loci. *CRISPR Journal* 2020;**3**(6):462–9. https://doi.org/10.1089/CRISPR.2020.0059;REQUESTEDJOURNAL:JOURNAL:CRISPR;WGROUP:STRING:PUBLICATION.

16. Camargo AP, Nayfach S, Chen IMA *et al.* IMG/VR v4: an expanded database of uncultivated virus genomes within a framework of extensive functional, taxonomic, and ecological metadata. *Nucleic Acids Res* 2023;**51**(D1):D733–43. https://doi.org/10.1093/NAR/GKAC1037.

17. Schmartz GP, Hartung A, Hirsch P *et al.* PLSDB: advancing a comprehensive database of bacterial plasmids. *Nucleic Acids Res* 2022;**50**(D1):D273–8. https://doi.org/10.1093/NAR/GKAB1111.

18. Yan M, Pratama AA, Somasundaram S *et al.* Interrogating the viral dark matter of the rumen ecosystem with a global virome database. *Nature Communications 2023 14:1* 2023;**14**(1):1–16. https://doi.org/10.1038/s41467-023-41075-2.
